# Fascialage: A Refined Technique of Dorsal Augmentation in Rhinoplasty

**DOI:** 10.1055/s-0045-1802554

**Published:** 2025-02-20

**Authors:** Prashantha Kesari, Sameer Halageri, Pradeep Kumar

**Affiliations:** 1 Department of Plastic Surgery, Nypunya Aesthetic Clinic, Bangalore, Karnataka, India

**Keywords:** augmentation rhinoplasty, fascialage, aesthetic rhinoplasty, rib graft for rhinoplasty, natural augmentation

## Abstract

**Introduction** The primary aim of this paper is to describe an original technique for dorsal augmentation in rhinoplasty. The use of a carved block of costal cartilage or layers of septal cartilage is a well-known technique for dorsal augmentation. However, this is accompanied by the risks of cartilage warping and a hard, unnatural feel to the dorsum. On the other hand, the use of diced cartilage wrapped in the fascia, which has been another popular technique in the last decade, often does not give the structural stability of a solid cartilage. “Fascialage,” as innovated by the authors, is a combined construct that incorporates all the advantages of these techniques eliminates the disadvantages of the previous techniques.

**Materials and Methods** Our technique involves the creation of a construct with three components and three parts. Radix augmentation is done with the cranial part, which consists of mostly rolled-up fascia; augmentation of the mid-dorsum is done with the central part, which has a solid cartilage platform, an overlay of diced cartilage with both being wrapped in the fascia, while the lower dorsum and supra-tip area also have all the same three components but with less diced cartilage. We can plan and alter the composition and dimensions as required for the individual case.

**Results** As a result of this, we believe that “fascialage” has the advantages of the other two techniques and consistent long-term results.

**Conclusion** This technique was used in 55 rhinoplasties needing considerable dorsal augmentation in which a postoperative follow-up of 2 to 5 years revealed that this technique yields reliable and sustainable results.

## Introduction

Aesthetic plastic surgery aims to achieve natural results by using tissues of similar physical characteristics. Since the dorsum of the nose has a unique composition and character, reconstruction or augmentation of this area, in patients who require it, needs to be a fine balance of stability and shape. It also must be sustainable and reproducible. A solid block of cartilage is stable, but the worry always is whether it will warp with time. In our experience, it also has an unnatural hard feel and a sharply defined appearance of the nose. On the other hand, diced cartilage wrapped in fascia does not warp, but the construct is often not stable enough in the long term. Our “fascialage” technique combines the best of both techniques for augmentation of the dorsum of the nose: soft fascia to reshape the radix in continuity with a stable cartilage base with an overlay of diced cartilage wrapped in the fascia over the dorsum and supra-tip area of the nose.

## Materials and Methods

A total of 55 patients were operated with the fascialage technique at Nypunya Aesthetic Surgery center by the main author.

Thirty patients were females, while 25 were males.

Thirty-seven were primary rhinoplasties, while 18 were secondary rhinoplasties. Indications were the following:

- Aesthetic augmentation: 24.
- Cleft rhinoplasty: 9.
- Deviated nose: 8.
- Implant replacement: 3.
- Multiple revisions: 5.
- Postsurgical saddle: 6.

Age ranged between 19 and 62 years, with the average age being 29 years.

Follow-up period ranged from 6 months to 6 years, with the average follow-up being 3 years. The average duration of the surgery was 3 hours and 20 minutes.

### Surgical Technique

All patients needing considerable augmentation of the nasal dorsum were included in the study. The dimensions of the augmentation deemed necessary were precisely determined by preoperative morphing in life-size photo printouts in profile views and intraoperatively using calibrated retractors and autoclaved rubber sizers. The “fascialage” construct was then used to achieve accurate dorsum augmentation in all these surgeries.

**Fig. 1:**
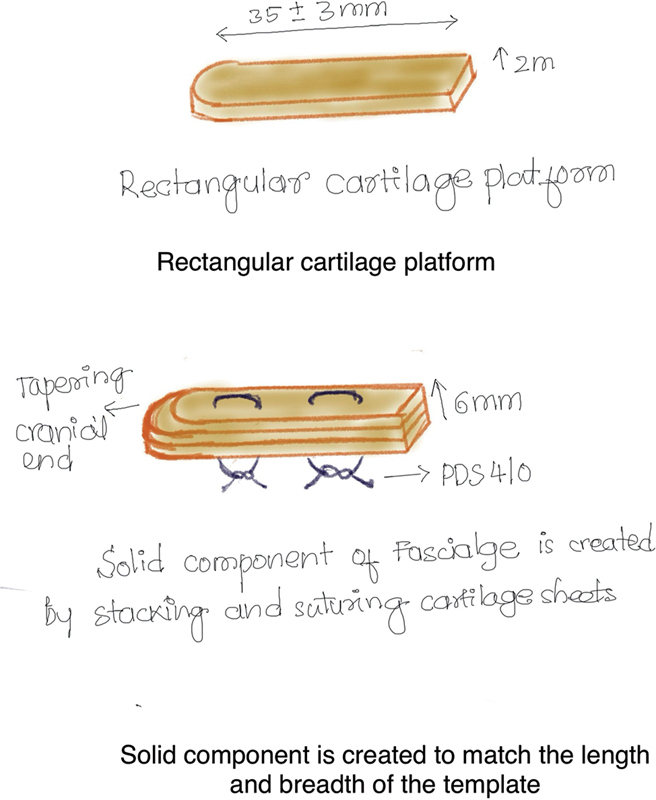
Cartilage solid base.

**Fig. 2:**
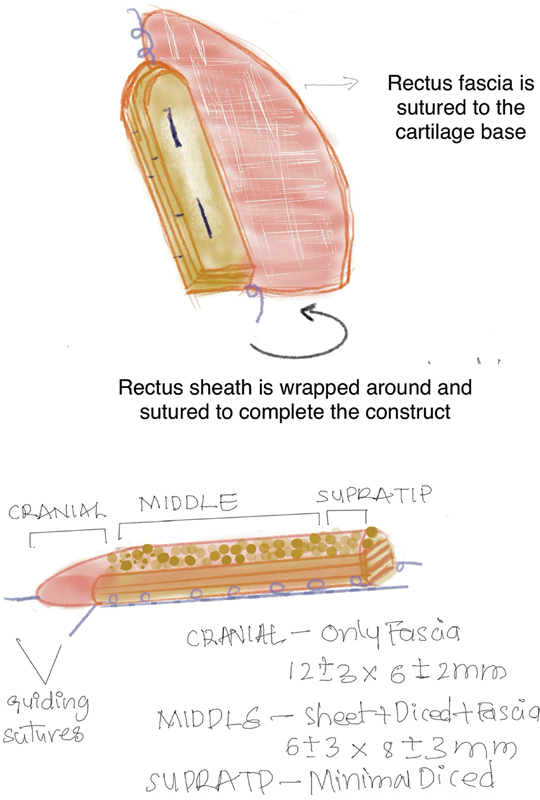
Fascialage making illustration.

“Fascialage” is a three-dimensional construct made of the fascia and cartilage with three parts and three components ( Figs. 1 and 2 ).

The three components are the following:

- A solid costal cartilage base platform.
- Rectus fascia, which is sutured to the platform to form a cylindrical pouch.
- Finely diced cartilage injected and packed as an overlay on the platform within the fascial • pouch.

The three parts are the following:

- Cranial part for the radix: fascia only.
- Central part for the dorsum proper: all the components.
- Caudal part for the supra-tip: all the components but with minimal diced cartilage.

### Harvesting the Components of the Fascialage Construct



**Video 1** Brief video of Fascialage Technique


With the patient under general anesthesia with endotracheal intubation, with the parts painted and draped, the rectus fascia and costal cartilage were harvested through a subpectoral incision in male patients and an inframammary crease incision in female patients.

Handheld ultrasound (eagle's view) was used to map the ribs precisely to find the uncalcified segments of cartilage, rectus fascia, and its interdigitations. Thickness of the subcutaneous layers is used to decide the incision length: the thicker subcutaneous layer, the longer the incision. Skin mobility from medial to lateral is an important factor in deciding the placement of the incision ( Video 1 ).

The sub-serratus anterior fascia nerve block with ropivacaine 0.25% under ultrasonography (USG) guidance ensures good preemptive anesthesia and postoperative pain relief.

Infiltration of the chest donor area is done using a solution of lignocaine and ropivacaine with adrenaline. An incision of length equal to subcutaneous layer thickness on USG (1.2–4 cm; average: 2.3 cm) was taken and deepened through the subcutaneous tissue to reach the rectus fascia by a combination of blunt and sharp dissections. A 2 cm ^2^ Scarpa fascia is harvested when well defined. A rectangular piece of the rectus fascia of 4 cm × 3 cm is harvested between the interdigitations. The rectus abdominis muscle is then split to reach the anteromedial surface of the ribs, previously decided by USG. The needle test is performed to check for calcification of cartilage. Usually the costal cartilage of the sixth or seventh rib from which a long and straight cartilage block of 4 to 5 cm in length and 8 to 10 mm in width with intact anterior perichondrium could be harvested was marked and carefully harvested. Hemostasis was confirmed. The donor area was checked for air leaks (pleural rents) and then closure was done meticulously in layers.

### Design and Execution of Fascialage

Through an open approach using a transcolumellar incision with infracartilaginous extensions, the entire nasal dorsum was carefully exposed in the subperichondrial up to the radix. An autoclaved rubber eraser carved to the size of the required construct in each individual case is used as an intraoperative sizer by which the dimensions (width and length) of the cartilage platform are assessed. A calibrated Aufricht retractor gives the dimensions to which the sizing rubber eraser needs to be cut. The carved eraser is then placed in the dorsum and skin redraped to assess the outcome.

Further modifications are done to the sizer till a satisfactory trial outcome is achieved. The eraser is then removed, and a thorough lavage is performed.

Handheld ultrasound gives us the dimensions of the uncalcified segments of costal cartilage. The decision to preserve the anterior perichondrium or to perform the oblique split method of Taştan et al depends on the dimensions of the harvested cartilage.

The harvested cartilage blocks had the anterior perichondrium left attached 2 to 3 mm wider and longer than the block size. The cartilage block was sliced by the oblique split method to rectangular slivers and were kept in saline to assess warping, as described by Taştan et al. 1 The other surface of the base cartilage platform was scored, both horizontally and vertically, to create a square pattern on the surface. We call this a “window pane” technique because it was inspired by the fact that longer window panes warp and smaller ones do not. The rectus sheath was sutured to the cartilage platform using continuous running sutures with 4–0 or 5–0 Vicryl.

When the cartilage segment is not long enough, we use rectangular 2-mm sheets of cartilage stacked and sutured to get the platform of required dimensions. In this technique, the rectus fascia is carefully sutured to the edges of the platform with 5–0 Vicryl.

The multiple small pieces of the cartilage block left after carving the base platform as well as some more harvested small pieces were then used as diced cartilage. They were finely diced with a skin grafting blade and then stuffed tightly/compressed into a 1-mL tuberculin syringe to get the desired consistency in the syringe. Small holes can be drilled in the syringe tip to help remove any fluid and any small air pockets within the compressed diced pieces.

The rectus fascia is rolled up on one end to form the cranial part, which will augment the radix. Below this level, on the dorsum, the construct will have all three components. The cartilage platform base has an intact perichondrium that extends 2 mm beyond the borders on all sides. This sleeve is now used to suture the rectus fascia to the platform/base, using 4–0 Vicryl, creating a closed three-dimensional fascial pouch with a solid base, in which an overlay of finely diced cartilage will be packed, injecting it through the Tuberculin syringe. This completes the construction of a composite graft, which matches the physical characters of a natural dorsum.

The composition of the solid cartilage platform is increased by adding additional layers of 2-mm-thick rectangular sheets, which are held together with mattress sutures of 4–0 PDS.

The proportion of solid component to diced component is decided by the thickness and rigidity of the skin envelope. A thick skin envelope needs a more solid component and vice versa.

The dimensions of the constructed fascialage are double checked. The fascialage is placed into the pocket over the dorsum with the help of three guiding sutures of 4–0 Vicryl at the top, brought out onto the skin. The middle one is brought out centrally between the eyebrows, while two lateral ones are brought out through the lower part of the radix.

The cartilage base platform, which sits on the dorsum in the area of the nasal bone and the upper lateral cartilage, is now secured with the help of PDS sutures at three points. Lower down on the dorsum, in the supra-tip area, the construct has the base platform, fascia, and minimally packed diced cartilage tapering smoothly. Tip work is then done as required and a trial closure of the open tip is done. Once the surgeon is satisfied with the shape and contours, the dimensions and position of the fascialage are rechecked and adjusted one final time before a definitive closure is done in layers using 5–0 Vicryl. The nose is taped and a splint is applied. The standard postoperative protocol used universally after any rhinoplasty is then followed.

Follow-ups were done after 1, 3, and 12 weeks, 6 months,1 year, and then yearly thereafter as far as possible.

The aesthetic parameters of the nose are assessed both subjectively and objectively.

## Results: Observations and Evaluation

- Mean incision length over the chest donor area for harvest: 22 mm.
- Mean operative time for constructing the fascialage: 40 minutes.
- Mean length of fascialage: 37 mm/cm.
- Mean width of fascialage: 67 mm.
- Mean height of fascialage: 72 mm ( Table 1 ).

**Table 1:** Clinical tabulation of procedures performed

Number	Indication	Primary	Secondary	Dimension	Incision	Duration
1	Revision		Yes	40 × 6 × 8	2	4
2	Augmentation	Yes		38 × 6 × 8	1.5	3
3	Cleft		Yes	43 × 7 × 10	2.5	5
4	Augmentation	Yes	–	36 × 6 × 10	1.5	3
5	Augmentation	Yes	–	40 × 6 × 9	1.75	4
6	Deviation	–	Yes	40 × 6 × 10	1.2	4
7	Augmentation	Yes	–	40 × 7 × 1	2	4
8	Deviation	–	Yes	40 × 7 × 9	3	5
9	Cleft BL	–	Yes	35 × 9 × 10	3	4
10	2 saddle	–	Yes	40 × 9 × 9	3	5
11	Augmentation	Yes	–	38 × 8 × 10	3	3
12	Augmentation	Yes	–	36 × 6 × 10	2	3
13	Augmentation	Yes	–	39 × × 7 × 9	2	3
14	Augmentation	Yes	–	35 × 8 × 9	3	4
15	2 implant replacements	–	Yes	37 × 6 × 9	2	4
16	Augmentation	Yes	–	36 × 6 × 9	2	4
17	5 redo surgeries	–	Yes	38 × 6 × 9	4	6
18	Augmentation	Yes	–	38 × 7 × 10	2	4
19	Augmentation	Yes	–	37 × 7 × 10	2	4
20	Cleft	Yes	–	36 × 6 × 7	2	4
21	Traumatic deformity	–	Yes	39 × 7 × 9	3	6
22	Redo	–	Yes	39 × 7 × 9	3	4
23	Redo	–	Yes	39 × 6 × 9	3	4
24	Cleft bilateral	–	Yes	35 × 7 × 8	2	4
25	Augmentation	Yes	–	38 × 6 × 8	2	4
26	Augmentation	Yes	–	39 × 7 × 9	2	4
27	Traumatic deformity	–	Yes	37 × 6 × 8	2	4
28	Traumatic deformity	–	Yes	36 × 6 × 7	2	4
29	Augmentation	Yes		36 × 6 × 8	2	4
30	Cleft	Yes	–	37 × 7 × 7	2	4
31	Binders	Yes	–	38 × 7 × 10	2	4
32	Augmentation	Yes	–	37 × 6 × 8	2	4
33	Traumatic deformity	Yes	–	36 × 6 × 6	1.2	4
34	Traumatic deformity	Yes	–	37 × 7 × 8	1.5	4
35	Submucous resection saddle	–	Yes	37 × 7 × 10	2	4
36	Augmentation	Yes	–	37 × 6 × 8	2	3
37	Implant replacement	–	Yes	38 × 7 × 9	2	5
38	Traumatic deformity	–	Yes	38 × 7 × 8	2	5
39	Cleft BL	Yes	–	39 × 8 × 12	3	6
40	Deviation	–	Yes	38 × 8 × 9	3	5
41	Traumatic deformity	–	Yes	35 × 7 × 8		
42	Binders	Yes	–	36 × 7 × 8	3	5
43	Cleft	Yes	–	36 × 7 × 8	2	4
44	Augmentation	Yes	–	36 × 6 × 7	1.5	3
45	Traumatic deformity	–	Yes	38 × 7 × 8	2	5
46	Implant replacement	–	Yes	37 × 7 × 8	3	4
47	Cleft	Yes	–	36 × 6 × 7	2	4
48	Augmentation	Yes	–	37 × 7 × 8	2	4
49	Cleft	Yes	–	36 × 7 × 7	2	4
50	Deviation	–	Yes	37 × 7 × 8	2	3
51	Augmentation	Yes	–	37 × 7 × 9	2	4
52	Deviation	Yes	–	36 × 7 × 8	1.2	5
53	SMR saddle	–	Yes	37 × 7 × 8	2	4
54	Augmentation	Yes	–	36 × 7 × 8	2	3
55	Augmentation	Yes	–	36 × 6 × 9	2	3

Postoperative evaluation was done at 3-month intervals/months by the operating plastic surgeon and an independent plastic surgeon, analyzing the following aesthetic parameters:

- Physical parameters:
  – Length.
  – Height.
  – Width of the dorsum and radix.
  – Dorsal aesthetic lines.
  – Secondary deviation or warping.

**Fig. 3:**
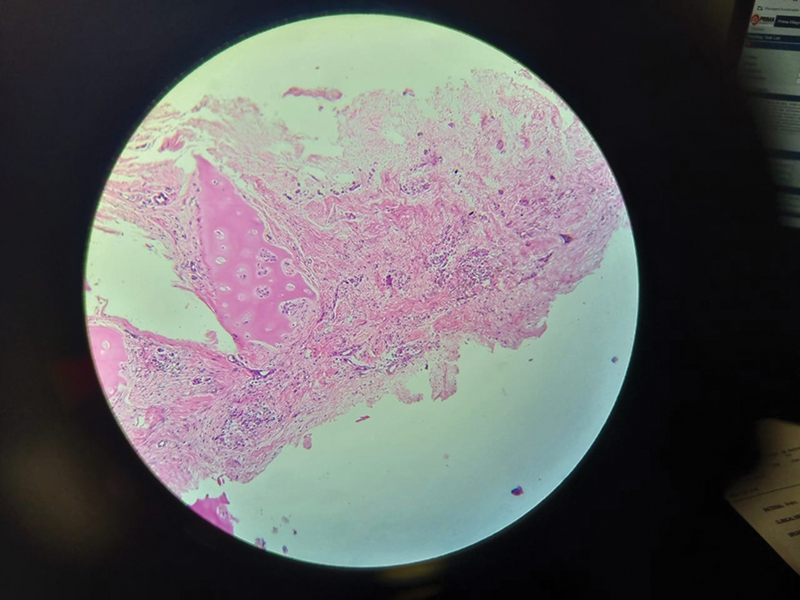
Histopathology showing viability of fascia and cartilage.

The length, height, and width measured at the time of surgery remained consistent at follow-up. The dorsal aesthetic lines were well maintained and smooth without obvious edges. No secondary deviation or warping was observed in any patient. No graft resorption, extrusion, and infection were observed. Overcorrection was observed in three patients and undercorrection in was observed in two patients. One patient underwent revision for over-augmentation at 12 months after surgery. A part of fascialage that was removed was sent for histopathological examination demonstrating viability of both fascia and cartilage ( Fig. 3 ).

- Tactile perception for consistency of the construct:
  – A natural feel of the dorsum was observed in all the patients. The hard feel generally encountered with a costal cartilage block graft was not present.
  – Persistent edema of the tip was observed in one patient beyond 6 months, which responded to intralesional triamcinolone.

**Fig. 4:**
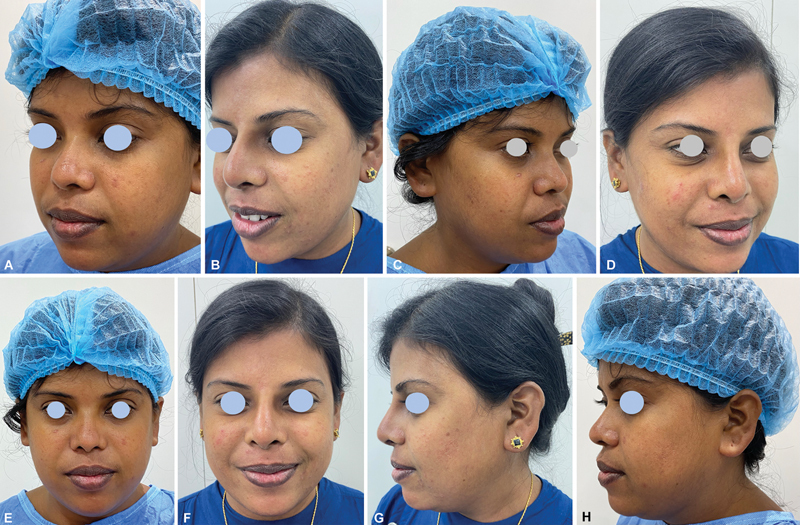
Nasomaxillary hypoplasia pre-op, ( **A** ) Nasomaxillary hypoplasia post-op ( **B** ). Nasomaxillary hypoplasia pre-op ( **C** ) and Nasomaxillary hypoplasia ( **D** ). Nasomaxillary hypoplasia post-op, ( **E** ) and Nasomaxillary hypoplasia post-op ( **F** ). Nasomaxillary hypoplasia pre-op, ( **G** ) Nasomaxillary hypoplasia post-op ( **H** ).

Patient satisfaction was evaluated with the 5-point Likert scale (show details of scale— very satisfied, satisfied, Neutral, dissatisfied, very dissatisfied). Fifty-one patients were very satisfied, two were satisfied, and the remaining two had a neutral opinion ( Figs. 4 5 6 ).

**Fig. 5:**
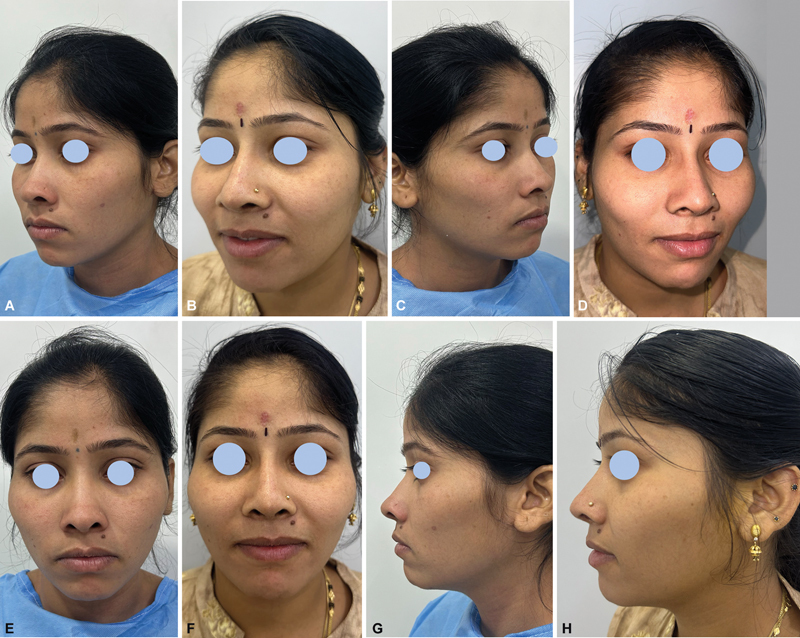
Dorsal augmentation pre-op, ( **A** ) Dorsal augmentation post-op. ( **B** ) Dorsal augmentation pre-op. ( **C** ) Dorsal augmentation post-op. ( **D** ) Dorsal augmentation pre-op. ( **E** ) Dorsal augmentation post-op. ( **F** ) Dorsal augmentation pre-op. ( **G** ) Dorsal augmentation post-op. ( **H** ).

**Fig. 6:**
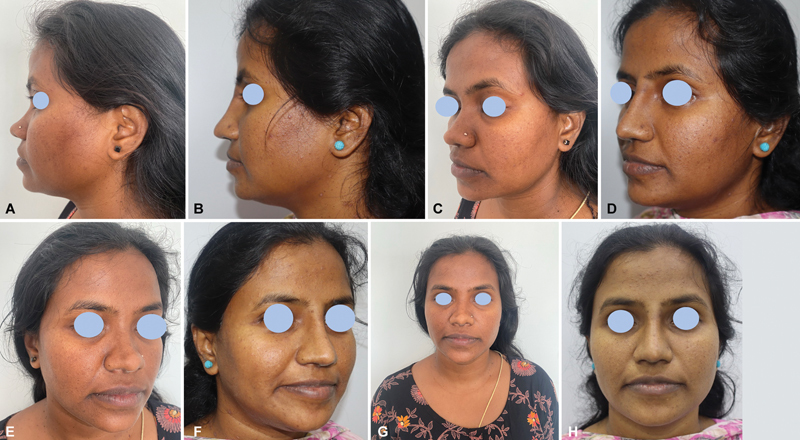
Moderate dorsal augmentation pre-op ( **A** ), Moderate dorsal augmentation post-op ( **B** ). Moderate dorsal augmentation pre-op. ( **C** ), Moderate dorsal augmentation post-op. ( **D** ), Moderate dorsal augmentation pre-op. ( **E** ) Moderate dorsal augmentation post-op. ( **F** ) Moderate dorsal augmentation pre-op. ( **G** ), Moderate dorsal augmentation post-op. ( **H** ).

## Discussion

The history of dorsal augmentation during rhinoplasty dates back to the 18th century and early attempts were crude, with a wide assortment of everyday materials 2 used to increase the height of the nose. Through the years, surgeons have attempted to improve outcomes by utilizing a variety of autologous and alloplastic materials, including cartilage, bone, 3 4 5 fascia, 6 diced cartilage and fascia, 7 8 9 10 silicone, 11 12 13 and other alloplastic materials. All of these have had mixed results.

Autologous cartilage grafts have several advantages in terms of survival, shaping, and morbidity. However, warping and visibility of the graft contours can be a problem in the long term, particularly for costal cartilage grafts. Revision rates for large solid dorsal onlay grafts carved from the rib cartilage are fairly high, even with the most experienced surgeons. 14

The complications observed with the use of solid onlay cartilage grafts led to the concept of using “diced cartilage,” which was considered an easier and more practical alternative for dorsal augmentation. The first documented use of diced cartilage in dorsal augmentation was as early as 1943 by Peer, 15 in 1951 by Cottle, 16 and in 1968 by Burian, 17 though it did not gain widespread acceptance at the time. Erol demonstrated the use of rolled Surgicel as a wrapping material over diced cartilage in 2000. 7 Daniel subsequently created a renewed interest with his description of wrapping diced cartilage in the fascia. 14 18 The choice of the fascia to be used also changed from the temporal fascia to the rectus fascia as described by Cerkes and Basaran, since it lies in the same donor area as the cartilage and could be harvested through the same incision, rather than a separate incision in the scalp. 19 20

Other modifications of the same concept of using diced cartilage as the building block for dorsal augmentation have been variously described, primarily adding tissue adhesives to ease shaping of the graft or altering the material wrapping the cartilage. 10 20 21 22 23 The main disadvantage of diced cartilage graft is that it does not give good structural support, and it cannot be used where a large amount of augmentation is required.

We have therefore designed a technique to club the advantages and minimize the disadvantages of autologous cartilage blocks and diced cartilage wrapped in the fascia. We have named this technique the “fascialage technique” for augmentation rhinoplasty.

Fascialage was also used to augment the premaxilla and the chin in cases of cleft and Binder's syndrome.

We believe that the great advantage of our technique is that it gives more structural stability and sustained form because of the solid sheet of cartilage that forms the base platform, as well as abundance of diced cartilage, which can be very nicely molded within the fascial wrap for desired augmentation.

The drawbacks of our study appear to be the following:

- An increase in the operative time to construct the fascialage.
- The need for a more precise analysis of the dimensions of augmentation.
- A longer follow-up to further confirm the advantages of this technique over the other commonly practiced techniques.

## Conclusion

The “fascialage technique” offers an ideal method of dorsal augmentation in rhinoplasty. The concept of this technique borrows the advantages of previous methods without their drawbacks. Since this technique does not need to harvest a full block of cartilage, the length of the donor site incision required, donor site morbidity, and postoperative pain are significantly less. It is possible to harvest the required size of the rectus fascia in between the interdigitations through the same incision.

Fairly accurate assessment of the dimensions of the augmentation required can be made with the help of intraoperative rubber sizers and calibrated retractors. The strong cartilage base platform shaped with the window pane technique gives good structural support without the risk of warping. The packed overlay diced cartilage fascia component gives an augmentation that has a natural feel and a stable form. Our “fascialage” technique can be used for augmentation even when the volume requirements are large. Our 2 to 4 years of follow-up so far has shown consistent and sustainable results. The fascia and diced cartilage take up vascularity well as demonstrated by histopathology.

Fascialage can be confidently used to get accurate augmentation of the dorsal of the nose.
