# Supplementary figures and images for: Fascialage: A Refined Technique of Dorsal Augmentation in Rhinoplasty
Source: Indian J Plast Surg. 2025 Feb 20;58(3):183–90. doi: 10.1055/s-0045-1802554 (PMC12213024; doi:10.1055/s-0045-1802554)

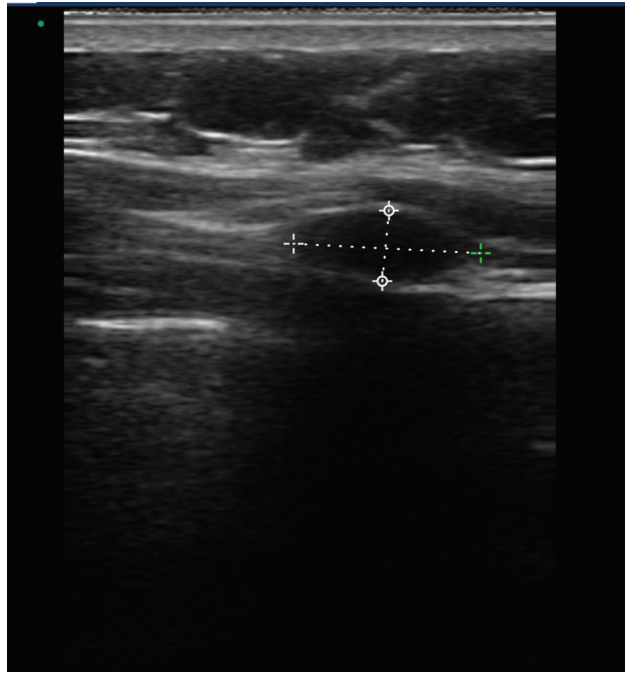

Supplementary Fig. 1 Ultrasound in planning rib harvest.

Supplement: Supplementary file 2 — Supplementary Material [file 10-1055-s-0045-1802554-s2422650.pdf]
